# Maternal high-protein/low-glycemic-index diet during pregnancy impairs offspring lipid profile–a randomized controlled trial

**DOI:** 10.1007/s00394-026-04032-5

**Published:** 2026-07-08

**Authors:** Christina S. Mogensen, Faidon Magkos, Elizaveta Chabanova, Jannie W. Jensen, Nina R. W. Geiker, Christian Mølgaard

**Affiliations:** 1 Department of Nutrition, Exercise and Sports, Faculty of Science, University of Copenhagen, Rolighedsvej 26, 1958 Frederiksberg C, Copenhagen, Denmark; 2 Department of Radiology, Copenhagen University Hospital Herlev-Gentofte, Herlev, Denmark; 3 Dietetic and Clinical Nutrition Research Unit, Copenhagen University Hospital Herlev-Gentofte, Herlev, Denmark

**Keywords:** Dietary intervention, Pregnancy, Offspring health, Offspring metabolic profile, Prevention of childhood obesity

## Abstract

**Purpose:**

We aimed to investigate the effects of a high protein content and a low glycemic index (HPLGI) diet during pregnancy on offspring body composition and metabolic health at 9 years of age.

**Methods:**

We conducted a randomized controlled trial in pregnant women with a pre-pregnancy BMI of 28–45 kg/m^2^ who were randomly assigned to either an HPLGI diet or a moderate-protein, moderate-glycemic-index (MPMGI) diet. Of the 208 live-born offspring, 114 were followed up at 9 years of age. Offspring blood samples were collected following an overnight fast, and continuous glucose monitoring data were obtained for up to 14 days. Body composition was assessed using dual-energy X-ray absorptiometry and magnetic resonance imaging.

**Results:**

Offspring in the HPLGI group exhibited 0.30 mmol/L (*P* = 0.033) higher total cholesterol, 0.24 mmol/L (*P* = 0.031) higher LDL-cholesterol, and 0.03 g/cm^2^ (*P* = 0.017) higher bone mineral density than those in the MPMGI group. LDL-cholesterol trajectories from birth to 9 years of age indicated that group differences in LDL-cholesterol increased with age and manifested later in life. Glucose homeostasis and adipose tissue mass did not differ significantly between groups.

**Conclusion:**

An HPLGI diet during pregnancy may negatively affect offspring total cholesterol and LDL-cholesterol. This underscores the need to investigate the long-term consequences of high-protein diets and the underlying mechanisms.

**Supplementary Information:**

The online version contains supplementary material available at 10.1007/s00394-026-04032-5.

## Introduction

The global prevalence of obesity in childhood and adulthood is increasing, leading to a higher number of women with obesity in the reproductive age [1, 2]. Pre-pregnancy obesity increases the risk of hyperglycemia, excessive gestational weight gain (GWG), gestational diabetes mellitus (GDM), and adverse infant outcomes, such as large-for-gestational-age (LGA) [3–5]. Offspring born LGA have an increased risk of developing obesity during childhood and adulthood [6, 7]. Moreover, there is an increased risk of childhood obesity when mothers have pre-pregnancy obesity [8, 9], but excessive GWG increases the risk of obesity throughout childhood, independent of pre-pregnancy BMI [9].

Intervention studies during pregnancy typically examine the effects of dietary manipulations for limiting GWG among women with pre-pregnancy overweight or obesity [10]. Maternal diets with a low-glycemic index limit GWG and reduce the risk of LGA infants among pregnant women with obesity [11, 12]. Moreover, a low-glycemic index diet has been shown to help regulate maternal fasting glucose and HbA1c homeostasis, contributing to improved glycemic control during pregnancy [13]. This not only enhances maternal metabolic health, but maternal fasting glucose is found to improve fetal growth and long-term metabolic outcomes in the offspring [14–17].

High protein intake in combination with low-glycemic index (HPLGI) foods is beneficial for weight loss and weight maintenance among non-pregnant individuals [18], and protein itself is found to be effective in limiting GWG [19]. However, observational and animal studies have found positive associations between maternal protein intake during pregnancy and offspring obesity risk, and unfavorable metabolic outcomes [20–22]. Impaired metabolic health during early childhood may be a risk factor for developing obesity later in life. In the APPROACH study, women consuming an HPLGI diet during pregnancy had fewer pregnancy complications and lower GWG than women consuming a moderate-protein, moderate glycemic index (MPMGI) diet [23]. However, offspring in the HPLGI diet had a worse lipid profile at 3 and 5 years of age compared with the MPMGI group, with no difference in birthweight and BMI z-scores from birth to 5 years of age [24].

Here, we investigated the effects of an HPLGI diet during pregnancy on offspring body composition and metabolic health at 9 years of age.

## Methods

### Study design

Pregnant women were cluster-randomized in groups of 4–8 women in gestational week (GW) 15 to either an HPLGI diet or an MPMGI diet. The women in the HPLGI group were guided to consume a diet high in protein, corresponding to 25% of total energy intake, and with a glycemic index of less than 55. The MPMGI group was guided according to the Nordic Nutrition Recommendations of approximately 18% protein, with no restrictions on glycemic index [25]. To support adherence to the respective ad libitum diets, participants in both groups received up to nine dietary counseling sessions, including two individual sessions and seven group-based sessions. To further assess adherence to the dietary protocol, we conducted sensitivity analyses using an objective biomarker of protein intake based on urinary urea excretion measured in GW 15, 28, and 36. Offspring of the participating women were followed up until the age of 9 years.

### Study participants

The pregnant women in the APPROACH study were encouraged by the sonographer at their first-trimester scan (between 11 weeks + 2 days and 13 weeks + 6 days of gestation) to participate in the study. The women attended an oral information meeting, received written participant information, and signed an informed consent. Women were eligible to participate if they planned to give birth at the Copenhagen University Hospital Herlev, were 18 years or above, had a pre-pregnancy BMI between 28 and 45 kg/m^2^, a singleton pregnancy, consumed less than 14 alcoholic items per week, and had no drug abuse. Women were excluded if they had other diseases at screening or disorders that developed during pregnancy that could influence the intervention (e.g., GDM).

This study included offspring born to women participating in the APPROACH RCT from January 2014 to December 2017 at the Copenhagen University Hospital, Herlev-Gentofte, Denmark. The follow-up study of the offspring ended in May 2025. Offspring assessments at the 9 year follow-up were performed at the Department of Nutrition, Exercise, and Sports, University of Copenhagen, Denmark, and at the Copenhagen University Hospital, Herlev, Denmark. The study was conducted according to the Declaration of Helsinki guidelines and approved by the National Committee on Health Research Ethics in Denmark (H-3-2013-119).

### Offspring measurements

#### Anthropometry and body composition

Offspring height was measured on a wall stadiometer (Hultafors, Sweden) to the nearest 0.1 cm, and circumferences were measured using a non-elastic measuring tape to the nearest 0.5 cm. Head circumference was measured three times, and arm, waist, hip, and thigh circumferences were measured twice—the average was used for analysis.

Body weight was measured in the morning after an overnight fast (≥ 10 h), and after the child had emptied their bladder. Body weight was measured to 0.1 kg using the integrated scale of a bioimpedance analysis device (InBody 570, USA).

Dual-energy X-ray absorptiometry (DXA) scan with a Lunar Prodigy whole-body scanner (GE Healthcare, USA; Software: enCore, version 17 SP1) was performed with participants wearing light clothing, in a fasting state, and after they had emptied their bladders. Total body less head bone mineral content (BMC), total body less head bone mineral density (BMD), fat-free mass (FFM), and fat mass (FM) were measured. Fat-free mass index (FFMI) and fat mass index (FMI) were calculated by dividing FM and FFM by the square of height (m^2^).

Magnetic resonance (MR) imaging and spectroscopy were performed on a 3.0 T MR7700 MR-imaging system magnet (Philips Medical Systems, Best, the Netherlands) at the Copenhagen University Hospital, Herlev, Denmark, to measure visceral adipose tissue (VAT), subcutaneous adipose tissue (SAT), and liver fat content. SAT and VAT were assessed by a chemical shift encoding-based water-fat imaging (mDixon). VAT and SAT volumes were calculated from fat fraction maps obtained by six-echo 3D mDixon Quant MRI in a transverse section of 10 mm thickness acquired in the middle of the third lumbar vertebra (L3). The volumes of VAT and SAT were measured by automatic thresholding and manual segmentation using a 3D Slicer [26]. Liver fat content was measured by a single-voxel spectroscopy (PRESS). The spectroscopy volume of interest (20 mm × 20 mm x 20 mm) was positioned in the right lobe of the liver to avoid major blood vessels and intrahepatic bile ducts. The procedures were performed with a standard postprocessing protocol for the MR imaging system by an experienced senior MR physicist.

#### Blood pressure

Blood pressure was measured three times with an interval of one minute between measurements using a blood pressure monitor (A&D Medical UA-787Plus, Tokyo, Japan), with appropriate-sized cuffs. Blood pressure was measured in a quiet room in a lying position, after 5–10 min of rest. The participants were not allowed to talk during the measurements. Blood pressure and pulse rate were measured on the right arm. The first measurement was performed to familiarize the children with the device and the pressure exerted by the cuff on the arm. The average of the 2nd and 3rd measurements was used in the analysis.

#### Biological sampling and analyses

Fasting blood samples were collected on the examination day. Plasma was separated into lithium heparin tubes and stored in 2 mL cryotubes at −70 °C until analysis. Plasma glucose and low-density lipoprotein (LDL)-cholesterol, high-density lipoprotein (HDL)-cholesterol, total cholesterol, triglycerides, and C-reactive protein (CRP) were analyzed on a Pentra 400 (Horiba ABX SAS). All CRP values above 10 mg/L were taken as an indication of infection [27] and were excluded (*n* = 1), and measurements below the detection limit of 0.11 mg/L were set to 0.055 mg/L (*n* = 21). Hemoglobin was analyzed using the Sysmex XQ-320 hematology analyzer (Sysmex Corporation, Japan). Plasma insulin and C-peptide were measured using the Cobas e411 immunoassay analyzer (Roche Diagnostics).

#### Continuous glucose monitoring

Continuous glucose levels were estimated using a continuous glucose monitoring (CGM) device (FreeStyle Libre Pro iQ Sensor system), which measures glucose levels in interstitial fluid every 15 min. The CGM was attached to the designated area on the arm for up to 14 consecutive days. The study participants were blinded to the CGM data, and no subject interaction was needed. Time in range (TIR) was calculated as the percentage of time spent in a pre-set glucose target range of 3.9–10.0 mmol/L. Time above range (TAR) was defined as time > 10.0 mmol/L, and time below range (TBR) was defined as time < 3.9 mmol/L. One participant was excluded due to a technical error.

#### Questionnaires

Information about the pubertal stage of the children was self-reported by their legal guardian, typically the mother. Tanner Stage with explanatory text and illustrations of the five pubertal stages was used to assess the development of breasts (girls) or genitals and pubic hair (boys) [28]. Puberty was defined as entry into Tanner Stage 2.

### Statistical analysis

Data from offspring who participated in the follow-up at 9 years of age were analyzed according to the intention-to-treat principle. Analyses were performed using simple linear regression models to estimate differences between the groups. Normality of continuous variables was assessed using visual inspection of distributions. We did not adjust for cluster randomization, as there was no indication of heterogeneity within clusters between groups. Group differences for continuous variables were assessed using independent t-tests when normally distributed and summarized as median (Q1; Q3) otherwise. Pearson’s Chi-squared test with Yates’ continuity correction was utilized for categorical variables.

Offspring with LDL-cholesterol and HDL-cholesterol measurements at 9 years were included in the analysis of LDL and HDL trajectories. To look at the difference in offspring outcomes between the two groups, these trajectories were examined using linear mixed models with age and group as fixed factors and with random effects for subject ID, without formal adjustment for multiple comparisons. All analyses were performed in R (version 4.2.3; R Foundation for Statistical Computing). A two-sided *P*-value ≤ 0.05 was considered statistically significant.

## Results

**Fig. 1:**
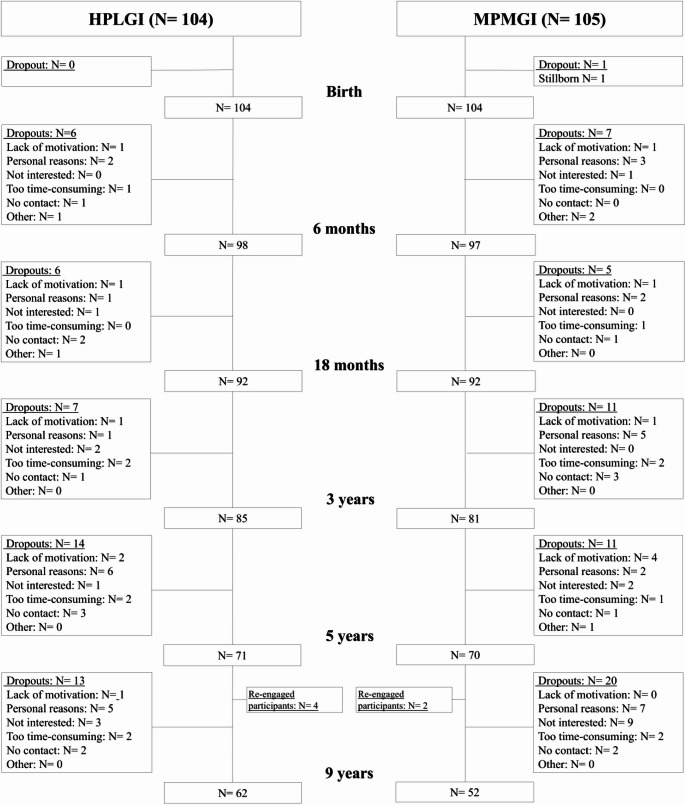
Participant flow and retention from birth to 9 years in the high protein low glycemic index (HPLGI) group and in the moderate protein moderate glycemic index (MPMGI) group. *N*, number of participants

A total of 209 children (HPLGI *n* = 104, MPMGI *n* = 105) were born to the women participating in the dietary intervention during pregnancy. One stillbirth occurred in the MPMGI group, leaving 104 participants in each group at birth. The dropout rate was 45.5% from birth to the follow-up visit at 9 years. At that time, 62 children remained in the HPLGI group and 52 in the MPMGI group (Fig. 1). The mothers in the HPLGI group had similar characteristics but gained 1.21 kg less body weight (*P* = 0.046) compared with the MPMGI group during pregnancy. Moreover, mothers in the HPLGI group consumed 25% energy from protein, significantly more than the MPMGI group, who consumed 18.9% energy from protein (*P* < 0.001) during pregnancy. Glycemic index was ~ 45 in the HPLGI and ~ 54 in the MPMGI, respectively (*P* < 0.001) (Table S1). There was no difference in maternal biomarkers during pregnancy, breastfeeding characteristics, and lifestyle factors at 9 years between the groups (Table S2, Table S4, Table S5). However, offspring from the HPLGI group exhibited higher glucose levels at birth compared with offspring from the MPMGI group (Table S3).

### Offspring cardiometabolic risk factors and body composition

Children in the HPLGI group exhibited 0.30 mmol/L (*P* = 0.033) or 7.4% higher levels of total cholesterol, 0.24 mmol/L (*P* = 0.031) or 11.3% higher levels of LDL-cholesterol, and a higher LDL/HDL-cholesterol ratio of 0.20 (*P* = 0.039) at 9 years of age compared with children in the MPMGI group (Table 1). HDL-cholesterol levels did not differ (Table 2).

**Table 1:** Anthropometry and body composition

	*N*	HPLGI	*N*	MPMGI	HPLGI-MPMGI	*P*-value
Age, years	62	8.75 (8.10;9.03)	52	8.41 (7.74;8.95)	0.15 (−0.09;0.39)	0.209
Sex, female, *n* (%)	62	24 (38.71)	52	21 (40.38)		1.000
Puberty girls, *n* (%)	23	8 (34.78)	20	7 (35.00)		1.000
Puberty boys, *n* (%)	36	3 (8.33)	31	2 (6.45)		1.000
*Anthropometry*						
Weight, kg	62	33.43 (8.02)	52	31.75 (6.98)	1.68 (−1.14;4.50)	0.240
Height, m	62	136.17 (5.94)	52	134.91 (7.11)	1.25 (−1.17;3.67)	0.307
BMI, kg/m^2^	62	17.84 (3.06)	52	17.31 (2.77)	0.53 (−0.56;1.63)	0.335
BMI-for-age	62	0.75 (1.25)	52	0.55 (1.24)	0.20 (−0.27;0.66)	0.403
Weight-for-age	62	1.13 (1.22)	52	0.96 (1.10)	0.18 (−0.26;0.61)	0.421
Height-for-age	62	1.05 (0.87)	52	0.98 (0.94)	0.06 (−0.27;0.40)	0.705
Head circumference, cm	62	53.87 (1.71)	52	53.91 (2.20)	−0.04 (−0.77;0.68)	0.910
Arm circumference, cm	62	21.90 (3.98)	52	21.04 (2.92)	0.85 (−0.33;2.04)	0.157
Waist circumference, cm	62	64.34 (9.51)	52	62.32 (8.01)	2.02 (−1.28;5.32)	0.227
Hip circumference, cm	62	73.19 (7.87)	52	71.55 (7.47)	1.64 (−1.23;4.50)	0.260
Thigh circumference, cm	62	40.61 (5.50)	52	39.40 (4.76)	1.21 (−0.72;3.14)	0.217
Waist-to-hip, ratio	62	0.88 (0.05)	52	0.87 (0.04)	0.01 (−0.01;0.02)	0.391
*Body composition (DXA)*						
Total bone mineral content, g	60	840 (137)	51	797 (132)	43 (−8.40;93.50)	0.101
Total bone mineral density, g/cm^2^	60	0.70 (0.06)	51	0.67 (0.05)	0.03 (0.00;0.05)	**0.017**
Fat-free mass, kg	60	22.65 (3.05)	51	22.22 (2.98)	0.44 (−0.70;1.58)	0.447
Fat mass, kg	60	11.11 (5.51)	51	10.02 (4.75)	1.09 (−0.86;3.05)	0.270
Fat-free mass index, kg/m^2^	60	12.17 (0.89)	51	12.14 (1.01)	0.03 (−0.34;0.39)	0.876
Fat mass index, kg/m^2^	60	5.87 (2.53)	51	5.40 (2.27)	0.47 (−0.44;1.38)	0.312
*Body composition (MR)*						
Subcutaneous adipose tissue, cm^3^	51	85.25 (65.27)	48	74.42 (56.01)	10.84 (−13.49;35.17)	0.379
Visceral adipose tissue, cm^3^	51	24.76 (13.07)	48	24.02 (12.28)	0.74 (−4.32;5.81)	0.771
Liver fat, %	50	0.82 (1.32)	47	1.09 (3.06)	−0.27 (−1.21;0.67)	0.568

Data are presented as means with standard deviation (SD) for continuous variables or as absolute and relative frequencies for categorical variables. DXA, Dual-Energy X-ray Absorptiometry; HPLGI, high protein low glycemic index; MPMGI, moderate protein moderate glycemic index; MR, Magnetic resonance. Different *N* values reflect occasional missing data for specific variables

**Fig. 2:**
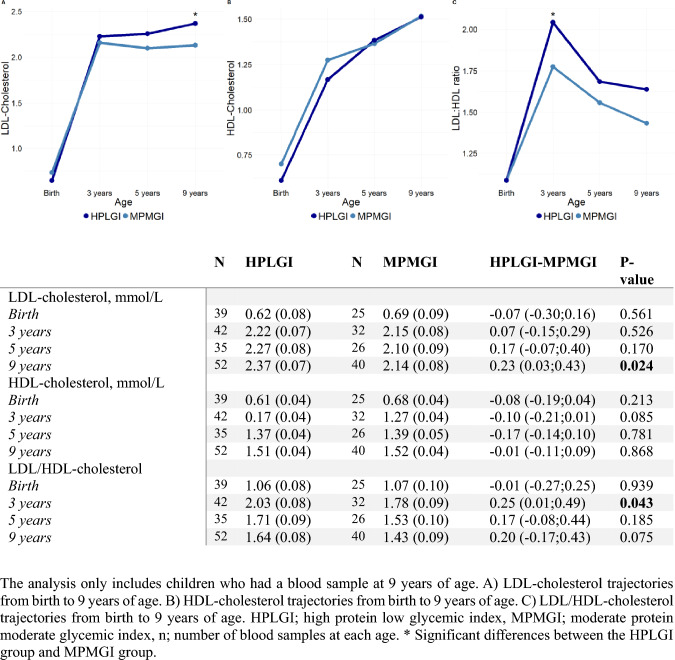
LDL-cholesterol and HDL-cholesterol trajectories from birth to 9 years of age

LDL-cholesterol and HDL-cholesterol trajectories from birth to 9 years of age revealed that changes in LDL-cholesterol manifest later in life and increase with time, whereas differences in HDL-cholesterol manifest early in life and decrease with time (Fig. 2). Accordingly, among those who completed the 9 year follow-up, HPLGI children had a significantly higher LDL/HDL-cholesterol ratio at 3 years of age. No significant differences were found for glucose homeostasis, blood pressure, and pulse (Table 2). However, the children in the HPLGI group had a higher BMD of 0.03 g/cm^2^ (*P* = 0.017) compared with the MPMGI group. The groups were similar in age, sex, pubertal stage, and anthropometric measurements at the 9 year follow-up (Table 1).

**Table 2:** Cardiometabolic risk factors

	*N*	HPLGI	*N*	MPMGI	HPLGI-MPMGI	*P*-value
*Blood pressure and Pulse*						
Systolic blood pressure, mmHg	61	101.88 (7.00)	52	104.94 (8.03)	−2.07 (−4.86;0.73)	0.147
Diastolic blood pressure, mmHg	61	66.84 (6.65)	52	66.98 (5.17)	−0.14 (−2.39;2.11)	0.899
Pulse, bpm	61	71.07 (9.56)	52	73.94 (8.15)	−2.88 (−6.22;0.47)	0.091
*Blood samples*						
Glucose, mmol/L	52	4.61 (0.29)	40	4.65 (0.36)	−0.04 (−0.18;0.09)	0.521
Hemoglobin, mmol/L	52	8.15 (0.40)	41	8.02 (0.45)	0.13 (−0.05;0.30)	0.151
Insulin, pmol/L	52	40.70 (39.85)	40	34.20 (19.93)	6.50 (−7.18;20.18)	0.348
C−peptide, ng/ml	52	1.28 (0.51)	40	1.18 (0.43)	0.10 (−0.10;0.30)	0.325
Total cholesterol, mmol/L	52	4.35 (0.72)	40	4.05 (0.58)	0.30 (0.02;0.58)	**0.033**
HDL-Cholesterol, mmol/L	52	1.51 (0.32)	40	1.52 (0.26)	−0.00 (−0.13;0.12)	0.936
LDL-Cholesterol, mmol/L	52	2.37 (0.57)	40	2.13 (0.44)	0.24 (0.02;0.45)	**0.031**
LDL/HDL-Cholesterol	52	1.64 (9.54)	40	1.43 (0.34)	0.20 (0.01;0.40)	**0.039**
Triglycerides, mmol/L	52	0.69 (0.30)	40	0.59 (0.25)	0.10 (−0.02;0.21)	0.104
CRP, mg/L	51	0.49 (0.68)	40	0.53 (0.87)	−0.04 (−0−36;0.29)	0.818
*CGM*						
Data obtained, n(days)	37	7.40 (3.90;11.10)	36	7.75 (4.15;10.23)	−0.36 (−2.31;1.60)	0.715
Active, %	37	99.67 (1.85)	36	99.60 (1.94)	0.07 (−0.82;0.95)	0.878
Mean glucose, mmol/L	37	5.62 (0.40)	36	5.55 (0.38)	0.07 (−0.11;0.25)	0.459
SD	37	13.46 (2.83)	36	14.41 (3.16)	−0.95 (−2.35;0.45)	0.180
CV	37	13.39 (2.95)	36	14.50 (3.33)	−1.11 (−2.58:0.36)	0.136
Time in range (3.9–10.0 mmol/L)	37	98.48 (2.96)	36	97.91 (3.88)	0.56 (−1.04;2.17)	0.486
Time above range (> 10.0 mmol/L)	37	0.03 (0.07)	36	0.07 (0.19)	−0.04 (−0.11;0.02)	0.205
Time below range (< 3.9 mmol/L)	37	1.49 (2.97)	36	2.02 (3.89)	−0.52 (−2.14;1.09)	0.520

Data are presented as means with standard deviation (SD). CGM, continuous glucose monitor; HPLGI, high protein low glycemic index; MPMGI, moderate protein moderate glycemic index. Bold indicates significantly different from the MPMGI group. Different *N* values reflect occasional missing data for specific variables

## Discussion

Our findings indicate that 9 year-old children born to women who consumed an HPLGI diet during pregnancy exhibited higher total cholesterol, LDL-cholesterol levels, and LDL/HDL cholesterol than offspring in the MPMGI group, suggesting a persistent alteration in lipid metabolism manifesting later in life (after birth).

Higher total cholesterol and LDL-cholesterol were also observed at 5 years of age, being 0.25 and 0.27 mmol/L, in HPLGI offspring than in MPMGI offspring, respectively [24]. Among the offspring who completed the 9-year follow-up, differences between the groups in LDL-cholesterol increased over time, whereas HDL-cholesterol showed the opposite pattern. The effect of an HPLGI diet during pregnancy creates an unfavorable lipid profile, evident by the significantly higher LDL/HDL ratio, which potentially increases the risk of cardiometabolic diseases in later life. A meta-analysis including randomized controlled trials comparing statin therapy with placebo among adults found that for every decrease of 1 mmol/L in LDL-cholesterol levels, there was a reduction of all-cause mortality and major vascular events by 12 and 21%, respectively [29]. Moreover, a reduction of total cholesterol was also linearly related to a lower risk of CVD [30]. We found 11.3% higher LDL-cholesterol levels and 7.4% higher total cholesterol levels in offspring from the HPLGI group compared with offspring from the MPMGI group, which may be clinically relevant for preventing cardiometabolic diseases, as lifelong exposure to higher levels of LDL-cholesterol is associated with increased cardiovascular risk.

The negative consequences of an HPLGI diet during pregnancy on offspring metabolic health are consistent with a rodent study reporting that offspring exposed to a high-protein maternal diet during pregnancy exhibited higher levels of total cholesterol and LDL-cholesterol than those exposed to a low-protein diet [21]. Another rodent study found that a maternal high-protein diet during pregnancy reduces the expression of insulin receptors and insulin receptor substrate 1 in the livers of adult female offspring, regardless of the post-weaning diet (i.e., food preferences and macronutrient composition of the diet) [31]. Furthermore, no differences were observed in fasting glucose homeostasis or lipid profiles, but there was a trend toward elevated insulin concentrations in offspring from the high-protein group compared with those from the low-protein group (*P* = 0.07) [31], which may indicate a compensatory mechanism with increased insulin secretion to preserve normoglycemia. While effective in the short term, chronic hyperinsulinemia is metabolically unfavorable and may predispose to insulin resistance later in life [32, 33].

We did not observe significantly higher insulin levels in the HPLGI group, nor significant differences in anthropometric measures or adipose tissue volume. However, the direction of the estimates suggested a tendency toward higher values among children in the HPLGI group, although these differences did not reach statistical significance. The higher body weight estimates may explain the observed higher BMD among children in the HPLGI group. Observational studies indicate that protein intake during pregnancy may influence fetal programming and increase the risk of developing obesity at later ages [20, 34]. Additionally, advancing age itself increases the likelihood of children developing overweight or obesity as they grow older [35]. In an analysis of observational data from the Danish National Birth Cohort, we found a higher body weight and BMI at 18 years of age in offspring exposed to an HPLGI diet during pregnancy [36]; however, these findings were not replicated in the present study at 9 years of age, which may be due to several factors, including differences in study design, sample size, and the younger age at follow-up in the current study. Moreover, a prospective cohort study found that elevated maternal protein intake was associated with increased offspring birth weight and a higher risk of overweight or obesity during childhood, particularly at ages 7 and 11 [37]. For every 5% increase in protein intake was associated with a 7 and 11% increased risk of childhood obesity at 7 and 11 years, respectively. These associations suggest potential developmental programming effects of maternal protein consumption becoming visible from the early peripubertal period.

The maternal diet can modify gene expression through epigenetic changes in lipid metabolism and adipose tissue development in the offspring; however, most animal studies have investigated the effect of a low-protein diet during pregnancy [38]. The higher LDL-cholesterol and total cholesterol levels, as well as the tendency toward higher adiposity in offspring from the HPLGI group, may therefore be influenced by potential epigenetic changes in lipid metabolism and adipose tissue function.

Despite the comprehensive data collection of cardiometabolic risk markers and lifestyle factors such as physical activity and diet, our study is not without limitations, as we are not able to provide a mechanistic explanation for the differences in cholesterol homeostasis between the HPLGI and MPMGI children. An important limitation of this study is the high attrition rate. Over a period spanning from pre-pregnancy to an offspring age of 9 years, nearly half of the original cohort was lost to follow-up. This may introduce selection bias and limit the generalizability of the findings, as the children who remained in the study could differ systematically from those who dropped out, even if the maternal characteristics did not differ. Furthermore, pubertal status was assessed by the parent, which may be subject to misclassification compared with clinical assessment. In addition, there was a considerable proportion of dietary data for the children at the 9-year follow-up that was missing, which may reduce the precision of the dietary estimates and limit the ability to fully explore the difference in dietary intake. Moreover, the RCT study was powered to detect a difference in maternal GWG, not offspring outcomes. Therefore, our study may lack statistical power to detect true effects on body composition and other lifestyle factors between the two groups. However, a major strength of the study is the comprehensive data collection, which included offspring dietary energy and macronutrient intake, physical activity, and sleep, as well as detailed body composition assessments using DXA and MR scans.

Taken together, these findings suggest that increased protein consumption during pregnancy leads to unfavorable changes in lipid metabolism and lipid profile later in offspring life, characterized by higher total cholesterol and LDL-cholesterol, which may be an early marker of increased cardiometabolic risk. Future longitudinal studies should examine whether these adaptations persist and contribute to the development of obesity, dyslipidemia, and insulin resistance during adolescence and adulthood.

## Supplementary Information

Below is the link to the electronic supplementary material.

Supplementary file1 (DOCX 68 KB)

## Data Availability

Data described in the manuscript, code book, and analytic code will not be made available due to confidentiality and ethical concerns. The data contains sensitive information requiring careful handling to protect participant privacy.

## Abbreviations

APPROACH: An optimized programming of healthy children
BMC: Bone mineral content
BMD: Bone mineral density
CGM: Continuous glucose monitoring
CRP: C-reactive protein
DXA: Dual-energy X-ray absorptiometry
FFM: Fat-free mass
FFMI: Fat-free mass index
FM: Fat mass
FMI: Fat mass index
GDM: Gestational diabetes mellitus
GW: Gestational week
GWG: Gestational weight gain
HDL: High-density lipoprotein
HPLGI: High-protein low-glycemic-index
LDL: Low-density lipoprotein
LGA: Large-for-gestational-age
MPMGI: Moderate-protein moderate-glycemic-index
MR: Magnetic resonance
RCT: Randomized controlled trial
SAT: Subcutaneous adipose tissue
TAR: Time above range
TBR: Time below range
TIR: Time in range
VAT: Visceral adipose tissue

## Declarations

### Clinical trial number

NCT01894139

### Clinical trial webpage

Study Details | NCT01894139 | An Optimized Programming of Healthy Children (APPROACH) | ClinicalTrials.gov
