# Supplementary material for: Maternal high-protein/low-glycemic-index diet during pregnancy impairs offspring lipid profile–a randomized controlled trial
Source: Eur J Nutr. 2026 Jul 8;65(5):203. doi: 10.1007/s00394-026-04032-5 (PMC13346312; doi:10.1007/s00394-026-04032-5)
Supplement: Supplementary file 1 — Supplementary file1 (DOCX 68 KB) [file 394_2026_4032_MOESM1_ESM.docx]

**Supplementary material for Mogensen et al. ”Maternal High-Protein/Low-Glycemic-Index Diet During Pregnancy Impairs Offspring Lipid Profile”**

**Christina Sonne Mogensen^1^**, Faidon Magkos^1^, Elizaveta Chabanova^2^, Jannie Wittrup Jensen^1^, Nina Rica Wium Geiker^1,3^, Christian Mølgaard^1^
^1^ Department of Nutrition, Exercise and Sports, Faculty of Science, University of Copenhagen, Copenhagen, Denmark. ^2^ Department of Radiology, Copenhagen University Hospital Herlev-Gentofte, Herlev, Denmark
^3^ Dietetic and Clinical Nutrition Research Unit, Copenhagen University Hospital Herlev-Gentofte, Herlev, Denmark.

##

**Corresponding author:** Christina Sonne Mogensen, Department of Nutrition, Exercise and Sports, Science, University of Copenhagen, Rolighedsvej 26, DK-1958 Frederiksberg C, Denmark, Phone: +45 3533 3284, Email: csm@nexs.ku.dk

Contents

[Methods 2](#_Toc230946273)

[Maternal characteristics and dietary intake 2](#_Toc230946274)

[Maternal biomarkers 2](#_Toc230946275)

[Offspring birth characteristics 2](#_Toc230946276)

[Offspring Dietary intake 3](#_Toc230946277)

[Offspring Accelerometer measurements 3](#_Toc230946278)

[Questionnaires 4](#_Toc230946279)

[Supplementary tables 5](#_Toc230946280)

[Supplementary Table 1: Maternal characteristics 5](#_Toc230946281)

[Supplementary Table 2: Maternal biomarkers 7](#_Toc230946282)

[Supplementary Table 3: Birth characteristics 8](#_Toc230946283)

[Supplementary Table 4: Breastfeeding characteristics in the two groups 9](#_Toc230946284)

[Supplementary Table 5: Lifestyle factors at 9 years of age 10](#_Toc230946285)

[References 11](#_Toc230946286)

# Methods

### Maternal characteristics and dietary intake

Maternal dietary intake during pregnancy was assessed using 24-hour dietary recalls conducted at gestational weeks 21 and 32, while glycemic index was collected using a 29-item food frequency questionnaire (FFQ) administered at gestational weeks 15, 28, and 36. The FFQ was validated against a 4-day weighed dietary record in a subgroup of 31 pregnant women with obesity (1). Estimated protein intake was calculated from urinary urea excretion in grams per day measured in 24-h urine samples.

Maternal height was measured to the nearest 0.5 cm by a wall-mounted stadiometer (Seca, Germany) at the screening visit. Pre-pregnancy weight was self-reported or obtained by the general practitioner in GW 6–8. Parity was obtained by questionnaires and categorized as 0 or ≥ 1. Gestational weight gain was calculated as the last measured weight before delivery minus pre-pregnancy body weight. Pre-pregnancy body mass index (BMI) was calculated as self-reported pre-pregnancy weight (kg) / height (m)^2^ at the screening visit, and categorized according to the World Health Organization (WHO) definitions, with normal weight referring to a BMI between 18.5-24.9 kg/m2, overweight between 25-29.9 kg/m2, and obesity as a BMI of 30 kg/m2 or above (2).

### Maternal biomarkers

Maternal venous blood samples were drawn in GW 15, 28, and 36 after an overnight fast (≥ 10 h) at the Copenhagen University Hospital Herlev-Gentofte. Maternal plasma glucose and plasma lipids (Total-cholesterol, HDL-cholesterol, and triglycerides) were measured on a Vitros 5.1 FS analyzer (Ortho-Clinical Diagnostics, Johnson & Johnson Medical, Denmark). LDL-cholesterol levels were calculated from Friedewald’s formula (3). C-peptide was measured on the Immulite 2000 Analyzer (Siemens; Siemens Medical Solutions Diagnostics, Germany).

### Offspring birth characteristics

Gestational age was determined from the crown-rump length measurement at the first-trimester scan.

Offspring length at birth was measured using a length measuring mat (ADE MZ10027-1) to the nearest 0.5 cm, and offspring body weight was measured by a medical scale (Tanita, Illnios, USA) to the nearest 10 g. Weight for length was calculated using the WHO standards for child growth (4). Offspring cord blood samples at birth were collected by the midwives. Cord blood glucose and lipids at birth were measured using a colorimetric method on a Pentra 400 (Horiba ABX SAS, France). Insulin, C-peptide, IGF-1, and IGFBP-3 were analyzed on Immulite 2000 (Siemens Healthcare, UK). Insulin levels < 14.4 pmol/L were analyzed using an ultrahigh-sensitive Insulin Elisa kit from Mercodia AB (cat no. 10-1132-01, lot no. 35009).

###

### Offspring Dietary intake

A 4-day weighted food record (WFR), three weekdays and one weekend day, was filled out by the parents using a self-administered web-based dietary assessment tool, myfood24®. The children’s parents were instructed verbally and viewed a video on how to use myfood24® on examination day at the Department of Nutrition, Exercise and Sports, University of Copenhagen. They were encouraged to weigh the food or use the pictures of portion sizes for estimations in myfood24®, if weighing was not possible. A written participant manual was offered to the participants on how to use myfood24®, and they were advised to contact project staff by email/message or call the project phone number if any further questions arose after the examination day. Participants who did not complete the 4-day WFR within two weeks were asked to register their intake as soon as possible the following days. Daily intake was calculated as the average of the four days. Myfood24® Danish database was compiled by Frida Fooddata v2 2017.20.02 (5), Livsmedeksverket, National Food Agency database v 2017.03.21 (6), and McCance, Widdowson v7, and UK-general brands plus Tesco (7).

### Offspring Accelerometer measurements

The ActiGraph GT3X-BT tri-axial accelerometer (ActiGraph, Pensacola, Florida, USA) was used to measure physical activity and sleep objectively. Data were analyzed using ActiLife 6.13. Participants were instructed to wear the accelerometer on the right hip for 24 h/day and were only allowed to take it off during water activities. Acceleration data were sampled at 30 Hz and downloaded as 60-second epochs (ActiLife6, ver. 6.11.4, Actigraph Corp., Pensacola, Florida, USA).

#### Physical activity

The “Troiano (2007)” algorithm provided by ActiLife6 was used to define non-wear-time, defined as a period of at least 60 consecutive minutes of zero counts, allowing for 2 min of non-zero interruptions (8). Moreover, sleep-period time was removed from the physical activity analyses by exclusion of sleep periods registered in the parent-reported sleep log for 8 days and 8 nights. A minimum wear time of ≥ 500 min/day was considered a valid day for the analysis, and at least 4 days (including at least three valid weekdays and one valid weekend day) were used as the criteria for a valid period (9, 10). To categorize the intensity of physical activity, previously validated cut-off points from Evenson et al. among children were used (11): Sedentary activity was classified as  ≤ 100 vertical counts per minute (cpm), light physical activity as 101–2295 vertical cpm, and MVPA as  ≥ 2296 vertical cpm.

#### Assessment of sleep

For analysis of sleep parameters, the ‘Sadeh’ sleep algorithm provided by ActiLife6 was used (12). In addition to the measurements, the parents were instructed to record the time when the child fell asleep and the wake-up time in the morning in a sleep log. We applied two different approaches to specify the night interval that was used for sleep scoring from the actograms. First, we used the sleep period in the sleep log. Second, we used visual inspection if the parents forgot to register sleep time, or the accelerometer was worn for more days than the sleep log. If sleep times were set by visual inspection of the actograms, the following criteria were used: The time when the child slept was defined as < 10 cpm after four consecutive minutes in the evening, and the wake-up time was defined as ≥ 10 cpm before four consecutive minutes in the morning. Nights where the monitor was reported to have been removed or nights with no recorded activity were excluded from the analyses of sleep (n = 26 nights). Likewise, nights with a sleep duration ≥ 15 h were excluded, as they may be considered as unusual behavior (e.g., sickness) (n = 0 nights).

### Questionnaires

Information on breastfeeding and the age of introduction to solid foods was collected through a questionnaire at 6 months of age. Breastfeeding duration was categorized into three time periods (0-2 months, >2-4 months, and >4-6 months).

# [Supplementary tables](#_Toc83807830)

# [Supplementary Table 1: Maternal characteristics](#_Toc83807831)

|  | N | HPLGI | N | MPMGI | (HPLGI-MPMGI Estimate) | P-value |
| --- | --- | --- | --- | --- | --- | --- |
| **Maternal age, years** | 62 | 33.75 (4.66) | 52 | 31.02 (4.74) | 0.03 (-1.72;1.78) | 0.974 |
| **Pre-pregnancy weight, kg** | 62 | 94.5 (11.92) | 52 | 96.15 (10.93) | 0.57 (-5.91;2.65) | 0.452 |
| **Height, cm** | 62 | 168.9 (5.8) | 52 | 167.4 (6.6) | 1.52 (0.77;3.81) | 0.192 |
| **Pre-pregnancy BMI, kg/m^2^** | 62 | 32.30 (30.62;34.79) | 52 | 32.81 (31.29;36.68) | -1.29 (-2.61;0.03) | 0.055 |
| **Pre-pregnancy BMI categories** |  |  |  |  |  | 0.109 |
| Overweight, % | 9 | 14.5 | 2 | 3.8 |  |  |
| Obese, % | 53 | 85.5 | 50 | 96.2 |  |  |
|  |  |  |  |  |  |  |
| **Gestational weight gain, kg** | 62 | 6.89±0.58 | 52 | 8.10±0.39 | -1.21 (-2.39-0.02) | **0.046** |
| **Nulliparous, %** | 61 | 54.1 | 52 | 57.7 |  | 0.815 |
|  |  |  |  |  |  |  |
| **Energy intake, kJ** |  |  |  |  |  |  |
| GW 21^1^ | 62 | 7501 (1942) | 51 | 7729 (1989) | -228 (-963;507) | 0.540 |
| GW 32^1^ | 60 | 7301 (2000) | 47 | 7497 (1929) | -197 (-957;564) | 0.609 |
| **Fat, E%** |  |  |  |  |  |  |
| GW 21^1^ | 62 | 33.0 (8.43) | 51 | 31.1 (8.00) | 1.88 (-1.20;4.97) | 0.229 |
| GW 32^1^ | 60 | 32.4 (8.41) | 47 | 32.4 (6.40) | 0.07 (-2.87;3.00) | 0.964 |
| **Carbohydrates, E%** |  |  |  |  |  |  |
| GW 21^1^ | 62 | 42.1 (8.49) | 51 | 49.9 (8.05) | -7.85 (-10.95;-4.74) | **<0.001** |
| GW 32^1^ | 60 | 41.6 (8.98) | 47 | 50.5 (7.48) | -8.85 (-12.08;-5.63) | **<0.001** |
| **Protein, E%** |  |  |  |  |  |  |
| GW 21^1^ | 62 | 24.9 (6.02) | 51 | 18.9 (5.00) | 6.00 (3.91;8.09) | **<0.001** |
| GW 32^1^ | 60 | 25.9 (5.82) | 47 | 17.1 (3.27) | 8.87 (6.99:10.75) | **<0.001** |
| **Glycemic index** |  |  |  |  |  |  |
| GW 15^2^ | 62 | 53.1 (4.84) | 52 | 53.7 (4.73) | -0.62 (-2.40;1.17) | 0.495 |
| GW 28^2^ | 61 | 44.1 (4.81) | 48 | 53.4 (4.24) | -9.25 (-11.00;-7.50) | **<0.001** |
| GW 36^2^ | 61 | 46.2 (4.65) | 45 | 54.5 (3.74) | -8.29 (-9.96;-6.62) | **<0.001** |
| **Estimated protein intake, g/day** |  |  |  |  |  |  |
| GW 15^3^ | 56 | 89.5 (22.61) | 47 | 94.5 (23.98) | -4.96 (-14.09;4.16) | 0.283 |
| GW 28^3^ | 60 | 99.6 (25.01) | 44 | 80.0 (19.34) | 19.61 (10.64;28.59) | **<0.001** |
| GW 36^3^ | 57 | 89.4 (22.36) | 42 | 72.8 (15.26) | 16.56 (8.62;24.50) | **<0.001** |

Table S1: Maternal characteristics. Values are mean (SD) for normally distributed values and median (Q1;Q3) for skewed distributions and estimate (95% CI) for between-group differences. Gestational weight gain was analyzed by using linear mixed models including time, group, and baseline maternal weight as fixed factors, and a group-by-time interaction with random effects of ID. Gestational weight gain is presented as adjusted means ± SE. E%, percentage of total energy intake. HPLGI, high protein low glycemic index; MPMGI, moderate protein moderate glycemic index. ^1^Data from 24-h recall. ^2^Data from FFQ. ^3^Estimated protein intake from urinary urea excretion. Different N values reflect occasional missing data for specific variables. Bold indicates significantly different from the MPMGI group.

# [Supplementary Table 2: Maternal biomarkers](#_Toc83807831)

|  | N | HPLGI | N | MPMGI | (HPLGI-MPMGI Estimate) | P-value |
| --- | --- | --- | --- | --- | --- | --- |
| **Glucose, mmol/L** |  |  |  |  |  |  |
| GW 15 | 62 | 5.0 (0.36) | 50 | 5.0 (0.39) | -0.02 (-0.16;0.11) | 0.774 |
| GW 28 | 61 | 4.9 (0.32) | 47 | 4.9 (0.40) | 0.02 (-0.12;0.15) | 0.801 |
| GW 36 | 59 | 4.9 (0.40) | 46 | 4.9 (0.37) | 0.01 (-0.13;0.16) | 0.848 |
| **C-peptide, pmol/L** |  |  |  |  |  |  |
| GW 15 | 61 | 507 (397;669) | 44 | 480 (380;584) | 22.27 (-52.72;97.26) | 0.557 |
| GW 28 | 60 | 643 (547;812) | 41 | 593 (482;915) | -60.84 (-206.93;85.25) | 0.411 |
| GW 36 | 59 | 868 (677;990) | 41 | 760 (569;944) | 43.66 (-92.51;179.83) | 0.526 |
| **Total cholesterol, mmol/L** |  |  |  |  |  |  |
| GW 15 | 62 | 5.10 (0.93) | 50 | 5.10 (0.72) | -0.01 (-0.33;0.31) | 0.960 |
| GW 28 | 61 | 6.10 (1.12) | 48 | 6.11 (0.91) | -0.01 (-0.40;0.39) | 0.969 |
| GW 36 | 60 | 6.50 (1.13) | 47 | 6.53 (1.26) | -0.04 (-0.50;0.42) | 0.872 |
| **Triglycerides, mmol/L** |  |  |  |  |  |  |
| GW 15 | 62 | 1.39 (0.46) | 50 | 1.43 (0.44) | -0.04 (-0.21;0.13) | 0.675 |
| GW 28 | 61 | 1.98 (0.56) | 48 | 2.07 (0.65) | -0.09 (-0.32;0.14) | 0.463 |
| GW 36 | 101 | 2.64 (1.08) | 92 | 2.86 (1.00) | -0.21 (-0.51;0.08) | 0.156 |
| **LDL-Cholesterol, mmol/L** |  |  |  |  |  |  |
| GW 15 | 62 | 2.91 (0.85) | 50 | 2.87 (0.64) | 0.05 (-0.24;0.33) | 0.749 |
| GW 28 | 61 | 3.50 (1.09) | 48 | 3.44 (0.87) | 0.07 (-0.32;0.45) | 0.735 |
| GW 36 | 60 | 3.67 (1.10) | 47 | 3.66 (1.16) | 0.02 (-0.42;0.45) | 0.935 |
| **HDL-Cholesterol, mmol/L** |  |  |  |  |  |  |
| GW 15 | 62 | 1.54 (0.30) | 50 | 1.59 (0.32) | -0.05 (-0.17;0.06) | 0.385 |
| GW 28 | 61 | 1.71 (0.38) | 48 | 1.74 (0.35) | -0.03 (-0.17;0.11) | 0.671 |
| GW 36 | 60 | 1.67 (0.40) | 47 | 1.66 (0.37) | 0.01 (-0.14;0.16) | 0.864 |

Table S2: Maternal biomarkers. Values are mean (SD) for normally distributed values and median (Q1;Q3) for skewed distributions with change and (95% CI) for between-group differences. HPLGI, high protein low glycemic index; MPMGI, moderate protein moderate glycemic index. Different N values reflect occasional missing data for specific variables.

# [Supplementary Table 3:](#_Toc83807831) Birth characteristics

|  | N | HPLGI | N | MPMGI | (HPLGI-MPMGI Estimate) | P-value |
| --- | --- | --- | --- | --- | --- | --- |
| Anthropometry |  |  |  |  |  |  |
| Gestational age, days | 62 | 279 (14) | 52 | 278 (20) | 0.98 (-5.30;7.25) | 0.759 |
| Weight, g | 62 | 3538 (408) | 52 | 3529 (739) | 8.7 (-208;226) | 0.937 |
| Length, cm | 62 | 51.86 (2.07) | 52 | 51.51 (3.66) | 0.35 (-0.73;1.44) | 0.519 |
| Weight-for-length | 62 | -0.75 (0.98) | 49 | -0.69 (1.39) | -0.06 (-0.50;0.39) | 0.807 |
| Blood samples |  |  |  |  |  |  |
| Glucose, mmol/L | 46 | 5.77 (1.41) | 28 | 5.10 (1.22) | 0.67 (0.03;1.31) | **0.041** |
| Insulin, pmol/L | 46 | 36.62 (31.12) | 27 | 48.28 (42.28) | -11.66 (-28.87;5.56) | 0.181 |
| C-peptide, pmol/L | 46 | 330.30 (166) | 28 | 341.50 (158) | -11-19 (-89.19;66.81) | 0.776 |
| Total cholesterol, mmol/L | 47 | 1.63 (0.43) | 28 | 1.79 (0.48) | -0.16 (-0.38;0.05) | 0.138 |
| HDL-Cholesterol, mmol/L | 47 | 0.64 (0.18) | 28 | 0.69 (0.18) | -0.05 (-0.14;0.04) | 0.250 |
| LDL-Cholesterol, mmol/L | 47 | 0.66 (0.24) | 28 | 0.72 (0.28) | -0.07 (-0.19;0.06) | 0.283 |
| Triglycerides, mmol/L | 47 | 0.58 (0.24) | 28 | 0.54 (0.28) | 0.05 (-0.08;0.17) | 0.461 |
| IGF-1, ng/mL | 46 | 55.77 (21.59) | 28 | 51.65 (21.38) | 4.13 (-6.15;14.41) | 0.426 |
| IGFBP-3, ug/mL | 46 | 1.69 (0.40) | 28 | 1.63 (0.33) | 0.06 (-0-12;0.24) | 0.507 |

Table S3: Birth characteristics Values are mean (SD) for normal distributed values and median (Q1;Q3) for skewed distributions with change and (95% CI) for between-group differences. HPLGI, high protein low glycemic index; MPMGI, moderate protein moderate glycemic index. Different N values reflect occasional missing data for specific variables. Bold indicates significant difference between groups.

# [Supplementary Table 4:](#_Toc83807831) Breastfeeding characteristics in the two groups

|  | N | HPLGI | N | MPMGI | P-value |
| --- | --- | --- | --- | --- | --- |
| Breastfed |  |  |  |  | 0.605 |
| Yes | 57 | 91.9 % | 49 | 96.1 % |  |
| No | 5 | 8.1 % | 2 | 3.9 % |  |
| Breastfeeding duration |  |  |  |  | 0.613 |
| 0-2 months | 7 | 11.3 % | 9 | 17.6 % |  |
| 2-4 months | 8 | 12.9 % | 5 | 9.8 % |  |
| 4-6 months | 42 | 67.7 % | 35 | 68.6 % |  |
| Solid foods |  |  |  |  |  |
| Introduction age, weeks | 56 | 20 (18;22) | 48 | 20 (16;21) | 0.372 |
| Introduction before week 16 | 12 | 21.4 % | 13 | 27.1 % | 0.658 |

Table S4: Breastfeeding characteristics in the two groups. Data are presented as mean (standard deviation (SD)) for continuous variables or as numbers (%) for categorical variables.

# [Supplementary Table 5:](#_Toc83807831) Lifestyle factors at 9 years of age

|  | **N** | **HPLGI** | **N** | **MPMGI** | **HPLGI-MPMGI** | **P-value** |
| --- | --- | --- | --- | --- | --- | --- |
| **Registration days** |  |  |  |  |  |  |
| Data obtained, days |  |  |  |  |  |  |
| Weekdays, n (%) | 49 | 132 (71.7) | 41 | 127 (73.0) |  |  |
| Weekends, n (%) | 49 | 52 (28.3) | 41 | 47 (27.0) |  | 0.884 |
| **Energy and macronutrient intake** |  |  |  |  |  |  |
| Energy, kcal | 49 | 1558 (388) | 41 | 1688 (434) | -130 (-302;42) | 0.137 |
| Energy, MJ | 49 | 6.52 (1.62) | 41 | 7.07 (1.82) | -0.54 (-1.26;0.18) | 0.138 |
| Carbohydrates, E% | 49 | 50.17 (5.88) | 41 | 49.00 (6.07) | 1.17 (-1.34;3.68) | 0.357 |
| Dietary fiber, g/MJ | 49 | 2.65 (0.72) | 41 | 2.62 (0.64) | 0.03 (-0.26;0.32) | 0.835 |
| Added sugar, g | 49 | 57.63 (22.59) | 41 | 59.36 (21.20) | -1.73 (-10.97;7.51) | 0.711 |
| Fat, E% | 49 | 32.22 (4.86) | 41 | 33.87 (5.27) | -1.66 (-3.78;0.46) | 0.124 |
| Protein, E% | 49 | 15.74 (2.82) | 41 | 15.22 (3.44) | 0.52 (-0.79;1.83) | 0.434 |
| **Intake of food groups** |  |  |  |  |  |  |
| Fruits, g/d | 49 | 131.40 (113.49) | 41 | 125 (81.74) | 5.80 (-36.40;47.99) | 0.786 |
| Vegetables, g/d | 49 | 151.48 (98.49) | 41 | 121.07 (59.22) | 30.42 (-4.49;65.32) | 0.087 |
| Wholegrains, g/d | 49 | 45.63 (35.30) | 41 | 53.58 (36.64) | -7.95 (-23.05;7.16) | 0.299 |
| Fish, g/d | 49 | 13.96 (18.34) | 41 | 8.36 (11.87) | 5.60 (-1.02;12.21) | 0.096 |
| Meat and meat products, g/d | 49 | 75.13 (46.52) | 41 | 84.16 (47.17) | -9.03 (-28.72;10.67) | 0.365 |
| **Physical activity** |  |  |  |  |  |  |
| Data obtained, days | 57 | 9 (8;9) | 49 | 9 (8;11) | -0.30 (-1.08;0.47) | 0.439 |
| Weekdays, n (%) | 57 | 380 (72.2) | 49 | 334 (71.5) |  |  |
| Weekends, n (%) | 57 | 146 (27.8) | 49 | 133 (28.5) |  | 0.855 |
| Sedentary time (min/day) | 57 | 378.38 (60.52) | 49 | 371.59 (63.57) | 6.79 (-17.14;30.72) | 0.575 |
| Light physical activity, min/day | 57 | 371.79 (48.63) | 49 | 389.57 (55.69) | -17.78 (-37.88;2.31) | 0.082 |
| MVPA, min/day | 57 | 51.13 (26.67) | 49 | 47.24 (23.80) | 3.89 (-5.92;13.70) | 0.434 |
| Total physical activity, cpm | 57 | 1166 (276) | 49 | 1149 (261) | 17 (-87;121) | 0.743 |
| Steps, min^−1^ | 57 | 13.19 (3.08) | 49 | 12.42 (2.91) | 0.77 (-0.39;1.93) | 0.191 |
| **Sleep** |  |  |  |  |  |  |
| Total sleep time, min | 57 | 560.90 (54.24) | 49 | 563.54 (54.28) | -2.53 (-9.53;4.47) | 0.478 |

Table S5: Lifestyle factors at 9 years of age. Data are presented as mean (standard deviation (SD)) for continuous variables or as numbers (%) for categorical variables. Between-group differences are presented as an estimate (95% CI). Cpm, counts per minute; HPLGI, high protein low glycemic index; MPMGI, moderate protein moderate glycemic index; MVPA, moderate to vigorous physical activity. Different N values reflect occasional missing data for specific variables.

# References

1. Mogensen CS, Vedelspang A, Geiker NRW. Validation of a food frequency questionnaire in the assessment of dietary glycemic index, glycemic load, and protein intake in pregnant women with obesity. Nutrition. 2024;118:112249.

2. Organization WH. Obesity and overweight 2024 [updated 1 March 2024. Available from: <https://www.who.int/news-room/fact-sheets/detail/obesity-and-overweight>.

3. Friedewald WT, Levy RI, Fredrickson DS. Estimation of the Concentration of Low-Density Lipoprotein Cholesterol in Plasma, Without Use of the Preparative Ultracentrifuge. Clinical Chemistry. 1972;18(6):499-502.

4. World Health Organization. WHO child growth standards : length/height-for-age, weight-for-age, weight-for-length, weight-for-height and body mass index-for-age : methods and development. Geneva: World Health Organization; 2006. vii, 312 p. p.

5. Frida Fooddata. Database Med Fødevaredata. Lyngby: DTU Fødevareinstituttet; 2024.

6. Livsmedelsverket (Swedish Food Agency). Uppsala: Livsmedelsverket (Swedish Food Agency); 2024.

7. Public Health England. McCance and Widdowson’s The Composition of Foods Integrated Dataset 2021. London: Public Health England 2021.

8. Troiano (2007) Wear Time Validation Parameters [Available from: <https://actigraphcorp.my.site.com/support/s/article/Troiano-2007-Wear-Time-Validation-Parameters>.

9. Hinkley T, O'Connell E, Okely AD, Crawford D, Hesketh K, Salmon J. Assessing volume of accelerometry data for reliability in preschool children. Med Sci Sports Exerc. 2012;44(12):2436-41.

10. Aadland E, Johannessen K. Agreement of objectively measured physical activity and sedentary time in preschool children. Prev Med Rep. 2015;2:635-9.

11. Evenson KR, Catellier DJ, Gill K, Ondrak KS, McMurray RG. Calibration of two objective measures of physical activity for children. J Sports Sci. 2008;26(14):1557-65.

12. Sadeh A, Sharkey KM, Carskadon MA. Activity-based sleep-wake identification: an empirical test of methodological issues. Sleep. 1994;17(3):201-7.
